# The role of infrared thermography in thyroid nodule detection and classification: a scoping review

**DOI:** 10.1007/s00405-026-10309-6

**Published:** 2026-06-01

**Authors:** Diego Filipe Bezerra Silva, Diana Lance, Elis Ângela Batistella, Scott Adams, Cassiano Francisco Weege Nonaka, Daniela Pita de Melo

**Affiliations:** 1 Post-Graduate Program in Dentistry, State University of Paraíba, R. Baraúnas, 351, Bairro Universitário, Campina Grande, PB 58429-500 Brazil; 2 College of Dentistry, University of Saskatchewan, Saskatoon, Canada; 3 University Medical Imaging Consultants, Medical Imaging, University of Saskatchewan, Saskatoon, Canada

**Keywords:** Thermography, Thyroid nodule, Thyroid diseases, Early detection of cancer, Skin temperature

## Abstract

**Purpose:**

To conduct a scoping review to assess the extent and type of evidence on the use of IRT as a diagnostic tool for thyroid nodule detection and classification.

**Methods:**

This review followed the JBI Scoping Review methodology and included studies involving IRT assessments of the thyroid gland in patients from clinical, academic, or experimental settings. Electronic searches were conducted in PubMed, Embase, Web of Science, Scopus, and Google Scholar.

**Results:**

A total of 28 studies published between 2009 and 2025 were included after screening 2,687 records. Most studies used static IRT (SIRT) or dynamic IRT (DIRT), many of them integrating artificial intelligence (AI) tools. The assessed studies demonstrated temperature differences between malignant nodules, benign nodules, and healthy tissues, particularly under thermal stress. AI classifiers, especially convolutional neural networks (CNN), have demonstrated enhanced diagnostic performance, with reported accuracies reaching up to 98.4%. However, results varied due to differences in imaging protocols, camera resolution, and patient-specific factors such as subcutaneous fat thickness.

**Conclusion:**

IRT shows promising potential as an adjunctive diagnostic tool for assessing thyroid nodules, particularly when combined with AI and dynamic protocols. However, standardization and further validation in diverse populations are needed.

**Supplementary Information:**

The online version contains supplementary material available at https://doi.org/10.1007/s00405-026-10309-6.

## Introduction

Thyroid cancer, the most frequent endocrine malignancy, comprises multiple histological subtypes that vary in prevalence and clinical aggressiveness [1]. The global incidence of thyroid cancer is significantly higher in women, occurring approximately three times more often than in men [2]. According to the Canadian Cancer Society, in 2024, 6,600 Canadians will be diagnosed with thyroid cancer, and 280 Canadians will die from thyroid cancer [3]. The American Cancer Society estimates 44,020 new cases of thyroid cancer in the United States for 2025 (12,670 in men and 31,350 in women), which are often diagnosed at a younger age than several other adult cancers (average age 51) [4]. As early detection of cancer improves survival rates and enables more cost-effective treatment [5], an easy and efficient screening system designed for the early detection of malignant thyroid nodules would benefit both patients and healthcare professionals. Such systems must also address the risk of overdiagnosis and be paired with improved classification methods to avoid unnecessary interventions.

US remains the most accessible and non-invasive method for thyroid assessment. Other imaging modalities such as magnetic resonance imaging (MRI), positron emission tomography (PET), scintigraphy, and computed tomography (CT) offer complementary information, particularly in complex cases involving retrosternal extension, metastasis, or functional characterization [6]. However, these methods involve higher costs, radiation exposure (CT, PET, scintigraphy), and often require contrast agents (CT, MRI), making them less suitable for routine assessment [7].

Malignant neoplastic nodules have intense cell proliferation, and they depend on blood vessels to obtain nutrients and oxygen, becoming highly vascularized structures [8]. Hence, some thyroid diseases induce temperature increase on the neck skin surface, which may serve as a potential diagnostic marker.^7^ Infrared thermography (IRT) offers a fast, painless, non-contact, non-invasive, and radiation-free method for photographically imaging temperature differences on the skin surface [9, 10]. Thus, it provides valuable insights for early detection of thyroid and lymph node abnormalities, especially inflammation, infection, or signs of hypervascularity (as seen in tumors). It may offer a quick and low-cost screening option for clinicians and potentially enable improved classification of nodules as benign or malignant [11].

IRT can be employed in two ways: without prior stimulus (static infrared thermography [SIRT]), or dynamically, by eliciting a response through the application of a thermal load (dynamic infrared thermography [DIRT]) [10, 12]. DIRT appears to improve IRT accuracy, primarily when combined with artificial intelligence (AI) [10]. Previous studies have shown wide variation in the accuracy of IRT for differentiating between malignant and benign lesions [10]. Therefore, this scoping review aims to assess the evidence on the use of IRT as a diagnostic tool for thyroid nodule detection and classification, facilitating early diagnosis, prognosis assessment, and timely treatment.

## Materials and methods

This scoping review was registered at the Center for Open Science, Open Science Framework (OSF) and followed the JBI Scoping Review Methodology Group protocol [13].

### Eligibility criteria

This scoping review aimed to answer the following question: “What is the extent and type of evidence on the use of IRT as a diagnostic tool for thyroid nodule detection and classification?”. This question was formulated following the Population (or participants)/Concept/Context (PCC) framework: **P-**Patients of any age, gender and ethnicity who have undergone IRT examination to assess the thyroid gland from private clinics, hospitals or universities; **C-**Assessment of the thermal camera parameters, temperature variations between normal and pathologic thyroid tissue, any techniques for improved detection or characterization (e.g., AI or DIRT); **C-**Subjective and objective data on thyroid nodule detection and thyroid cancer detection.

Scientific manuscripts and international conference extended abstracts of studies that assessed patients of any age, gender, and ethnicity who have undergone IRT examination to assess the thyroid gland from private clinics, hospitals or universities were selected for review assessment. Studies published between 2000 and 2025, and written in the Latin-Roman alphabet. Review and opinion papers, in vitro, and animal studies were excluded from this scoping review.

### Information sources and search strategy

A comprehensive list of search terms was derived, and MeSH (Medical Subject Headings) and non-MeSH terms were combined with Boolean operators “AND” and “OR” (Appendix I). An electronic search strategy was conducted in September 2025 using four databases (PubMed, Embase, Web of Science and Scopus). Additionally, Google Scholar was used to search for grey literature (the first 100 articles were considered). A manual search was also performed through systematic analysis of references of eligible articles. The detailed search strategy for each database is presented in Appendix I.

### Study selection and qualification

Study selection was conducted in two stages. First, titles and abstracts were independently screened by two reviewers against the predefined eligibility criteria. Full texts of potentially relevant articles were then assessed for inclusion. Disagreements were resolved through discussion or consultation with a third reviewer.

Although methodological quality appraisal was not performed, studies were required to meet minimum eligibility standards, including: (i) use of human participants, (ii) application of infrared thermography to the thyroid region, and (iii) reporting of original empirical data. Only studies providing sufficient methodological detail to allow extraction of technical, clinical, and analytical characteristics were included.

### Data items and synthesis methods

Two reviewers independently analyzed the studies and extracted the following information: year of publication, country, population, sample, age range, sex, study objective/target, thermal camera parameters and resolution, type of IRT (SIRT or DIRT), region of interest (ROI) selection method, algorithms, and main results and conclusions.

Due to clinical and methodological heterogeneity, a narrative synthesis of the results of main outcomes was carried out.

## Results

### Study selection

**Fig. 1:**
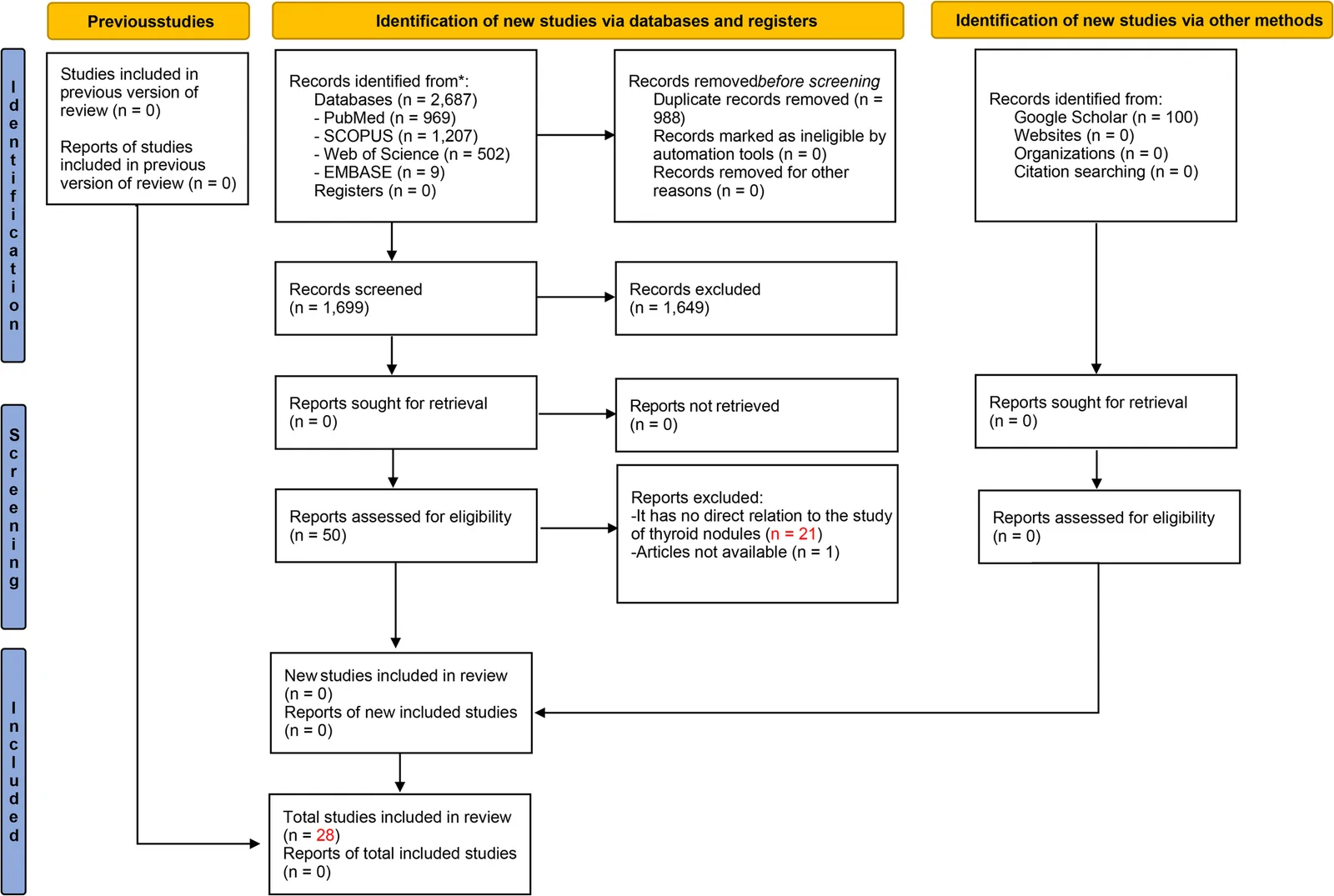
Flowchart of the search in the databases

The search strategy retrieved a total of 2,687 records. The first 100 results obtained by applying the search terms on Google Scholar were also reviewed. Of these 2,687 records, 988 duplicates were removed. The titles and abstracts of 1,699 studies were read, and 1,649 studies were excluded. Most studies were excluded due to their focus on animal tests, literature reviews, mathematical models, or assessments of other body areas, and published before the year 2000. Fifty records were considered potentially eligible, and their full texts were assessed by the two reviewers. After reading the full texts of the pre-selected articles, 22 studies did not meet the inclusion criteria. Therefore, the final sample consisted of 28 papers [5, 7, 8, 11, 12, 14–36]. The flowchart depicting the article selection process is shown in Fig. 1.

### Study characteristics

The included studies were published between 2009 and 2025. Most of the studies were carried out by authors linked to institutions in Brazil [5, 8, 12, 14, 15, 18–20, 25, 26, 31, 35], while four were associated with institutions in Iran [7, 21–23], three from Romania [28–30], two from USA [23, 24], two from Mexico [24, 27], one from India [32], one from Italy [33], one from Spain [24], one from Portugal [36], one from China [11], one from Turkey [16], and one from Thailand [17] (Table 1). Of the studies that reported the number of patients or exams studied, there was a wide variation, ranging from one patient [14, 20, 36] to 2,864 patients [11]. Information regarding the thermal camera was available in 24 studies. Of these, only one used a 1,280 × 720 resolution camera [32].

**Table 1:** Summary of the results from the studies evaluating the detection of malignancy, nodule identification (regardless of benignity), and recurrence

**Author/year/Country**	**Study objective/target**	**Population/sample/age range/sex/disease**	**Main results**	**Main conclusions**
***Detection of malignancy***				
Bahramian and Mojra, 2020, Iran	To compare the existing biomarker with the imaging modality for the early-stage diagnosis of thyroid disorder	18 patients (10 healthy and 8 cancerous cases)	Cancerous tumors caused 1–1.5°C local temperature rise; Disrupted symmetry in thermal maps; Good agreement (2–4% error) between tumor size in IRT vs CT images; 3D shape of tumor could be reconstructed from thermal data	IRT can localize and estimate the shape of vascular-phase malignant thyroid tumors; Proposed method allows non-invasive boundary detection; Strong potential for early cancer diagnostics and numerical modeling
Camargo et al., 2022, Brazil	To analyze thermal behavior of thyroid nodules using cold stress	33 patients with nodules (42 nodules - 11 malignant and 31 benign), 9 controls; sex/age not detailed	The Wilcoxon test showed thermal differences between groups (control and healthy, *p* < 0.001). The difference in the thermal behavior of the nodular tissues was evidenced by the longitudinal analysis. When comparing the nodules, it was possible to verify that the beginnings of tissue heating is significant (*p* = 0.001).	Benign nodules present a different thermal behavior than malignant nodules, and both present different behavior than normal tissue. For the analysis of nodules, the protocol used with cold stress, dynamic thermography and the inclusion of time t-1 were essential for the differentiation of nodules in the thyroid gland.
Chantasartrassamee et al., 2024, Thailand	To develop a diagnostic tool for thyroid cancer using infrared thermal images combined with an AI algorithm.	98 patients (mean age 55.3± 1.4 years and 78.6% F), 58 participants had malignant thyroid nodules (56 papillary thyroid carcinoma and 2 oncocytic thyroid carcinoma) and 40 had benign thyroid nodules.	AI model: 75% accuracy, 78% sensitivity	The infrared thermal images, assisted by an ai model, exhibit good performance in distinguishing thyroid malignancy from benign nodules. This imaging modality has great potential to be used as a noninvasive screening tool for adjunct evaluation of thyroid nodules
Damião et al., 2021, Brazil	To evaluate the temperature differences between benign and malignant TN by using IRT.	113 patients/malignant nodules (n = 27; 35–60 years; 70.4% F) and benign nodules (n = 120; 53–64 years; 95.8% F)	The ROC curve analysis of ΔT showed a cutoff of 2.38°C, with sensitivity of 0.963 and specificity of 0.992. Mt (33.46°C vs. 33.01°C, p < 0.0001), Ft (33.99°C vs. 33.38°C, *p* < 0.0001), and ΔT (2.95°C vs. 1.73°C, *p* < 0.0001) were higher in malignant nodules. Only It was lower in the malignant group (31.22°C vs. 31.68°C, p < 0.0001).	Malignant nodules have higher temperature than benign nodules on thermographic evaluation. These findings demonstrate that thermography could be a useful tool for the investigation of malignant TNs.
D’Arbo Alves et al., 2016, Brazil	To compare power Doppler US and thermography for the selection of thyroid nodules to be biopsied.	110 patients (98 F and 12 M); 124 nodules were analyzed (105 in F and 19 in M); Among the F, ages ranged from 18 to 68 years (mean, 50 years; median, 60 years), whereas they ranged from 18 to 78 years (mean, 67 years; median, 58 years) among the M; 15 cases of malignancy	Sensitivity, specificity, positive predictive value, negative predictive value, and accuracy of thermography were 100%, 58.06%, 87.73%, 100%, and 89.51%, respectively.	Thermography was more precise than was power Doppler US for the selection of thyroid nodules to be biopsied.
Etehadtavakol et al., 2025, Iran, Singapore, USA	To determine the most relevant features and assess the performance of different feature selection methods—unsupervised and supervised (including filter, wrapper, and embedded approaches)—in enhancing the classification accuracy of thyroid nodules from thermographic images.	Dataset consisted of 131 thermographic images, each corresponding to a distinct patient, comprising 108 benign and 23 malignant cases.	Within the feature selection approaches, LASSO yielded the highest performance, reaching 86% accuracy with an AUC of 0.91 for the Random Forest model and 86% accuracy with an AUC of 0.92 for the XGBoost model.	Feature selection significantly improves thyroid nodule classification in IRT. Random Forest and XGBoost achieved the best performance, especially with LASSO and variance thresholding, highlighting the IRT’s potential as a non-invasive complementary diagnostic tool.
González et al., 2019, Brazil	To show: 1) an analysis of static temperatures in the region of the nodule on the neck skin, 2) an analysis of temperatures series from thermograms captured dynamically, and finally 3) the importance of the correct selection of ROI to analyze the thyroid thermograms captured with DIRT protocol.	16 patients (9 with malignant nodules, 7 with benign nodules); age/sex not fully detailed	No consistent temp threshold differentiated benign vs. malignant nodules; Malignant regions not always hottest part of ROI; Cluster analysis (k-Means) did not clearly segment nodule regions unless k ≥ 5; Clavicle areas were often hotter and could skew results	Thermogram temperature ranges alone do not reliably distinguish malignant from benign nodules; Segmentation methods must focus on nodular regions, excluding hotter unrelated areas; Patient-specific temperature normalization and better ROI targeting are necessary for pattern identification
González et al., 2021, Mexico	To develop a thermal interpretation procedure, that could eventually become a standard in the detection of thermal anomalies in the neck which could point towards thyroid pathologies.	108 thermographies of thyroid pathologies	Values of ΔT ≥ 0.5 ºC are consistent with thyroid pathology and values of ΔT ≥ 1.0 ºC appear in the case of malignant thyroid pathology.	A thermographic interpretation procedure based on obtaining a ΔT value between a thyroid region of interest and its contralateral region could be used as a standard procedure for adjunct screening and analysis of thyroid pathologies.
Gavriloaia et al., 2009, Romania	To develop and assess a high spatial-temperature resolution mapping system for early cancer diagnosis, including thyroid cancer	Develop and assess a high spatial-temperature resolution mapping system for early cancer diagnosis, including thyroid cancer	Cancerous ROIs were more asymmetrical and ~1.5°C hotter than surrounding tissue; Non-cancerous ROI showed ~0.5°C difference; Thermistor offered superior temperature resolution (0.04°C) vs IR camera	High-resolution temperature maps (0.5 mm, 0.04°C) aid early thyroid cancer detection. Irregular, asymmetric thermal profiles indicate malignancy. The system achieved a 78.4% diagnosis rate, making thermography promising for pre-invasive diagnostics and monitoring.
Gavriloaia et al., 2009, Romania	To investigate IR signatures of thyroid tumors	24 patients with thyroid nodules	Malignant nodules show distinct, irregular signatures	IRT may help detect and differentiate malignant thyroid nodules
Gavriloaia et al., 2010, Romania	To evaluate whether fractal analysis of infrared thermal images of the neck could be used to differentiate thyroid diseases, and to explore its potential as a noninvasive tool for early diagnosis.	Papillary thyroid cancer and hyperthyroidism; Sample size/age/sex not fully detailed	The fractal dimension was 1,4217 for hyperthyroidism and 1,818 for papillary cancer. Lacunarity parameters also differed markedly, with β values of 3,624 and 28,581, respectively.	Self-similarity and lacunarity are two fractal parameters that can be used to assess thyroid diseases and distinguish between them. However, extensive research will be required in the near future to further develop this approach.
Moran et al., 2019, Brazil	Evaluate whether quantitative thermal features and CNNs can be used to classify thyroid nodules in thermographic images	Thermographies of 18 patients with malignant nodules; 85 patients with benign nodules; and 2 healthy patients, called the control group	The good results of CNN in the classification (with more than 92% of accuracy), show that the viability of the proposed methodology depends on the success of the segmentation	The tests show that the only feature that can be partially consider is the P_A_, since for most cases the P_A_ values for healthy areas are different to the values for nodules areas
Pathak et al., 2022, India, United Arab Emirates	To present an AI-based deep learning technique to detect cancerous nodules from thermograms for patients with hyperthyroidism.	Thermographic image dataset of thyroid region; exact patient/sample details not provided	The trained CNN has a classification accuracy of 91.11%, an error rate of 0.0975, a precision of 0.9167, and arecall value of 0.93076.	Thermal imaging may be applied as a learning method for the detection of thyroid nodules in the sample of thermal images. The developed method is prone to overfitting due to limited number of samples
Perpetuini et al., 2023, Italy	To examine the capability of IRT applied to the neck skin to distinguish TC, TA, and HC without the use of cold-stress protocols.	11 individuals (age: 65.76+−8.18 years; 6M) consisting of three participants in the TC group, four participants in the TA group, and four participants in the HC group.	The average temperature differences between the ROIs were 0.80°C (±0.26°C) for TC patients, 0.56°C (±0.29°C) for TA patients, and 0.09°C (±0.06°C) for HC. ANOVA showed significant differences for both maximum (p = 0.023) and average temperature (p = 0.002), with Bonferroni identifying significant differences between TC and HC.	The results indicated a distinct thermal pattern correlation with the presence and severity of thyroid cancer. These results could foster the employment of IRT for a non-invasive thyroid tumour diagnosis.
Salles et al., 2020, Brazil	To present semiautomatic segmentation software developed. It can analyze dynamic thermograms	Two thyroid tumors, one malignant and one benign	Malignant tumors consistently showed higher temperatures and larger segmented areas than benign nodules; Software allowed detailed tracking of temperature variation and region size over time; Enabled visual and graphical analysis distinguishing tumor types	The developed software improves speed, accuracy, and depth of dynamic thermogram analysis; Distinct thermal behaviors between benign and malignant nodules were detectable; Tool is effective for supporting biopsy decisions and clinical assessments in thyroid tumor diagnosis
Schadeck et al., 2020, Brazil	To develop a semiautomatic segmentation model of thermographic images using the Python computational language	Thermograms of 20 patients diagnosed with a tumor (benign or malignant), 8 of which were breast masses and 12 thyroid nodules	Semi-automatic method detected ROIs faster (avg. 16 s vs. 40 s manually); Average and minimum temperatures were more accurate (e.g., 1.1°C higher for thyroid nodules)	The developed software was able to segment the ROI of breast and thyroid thermograms. In addition, the proposed model comprises the tumor region with greater reliability than the manual ROI delimitation method.
Zhang et al., 2023, China	To present a novel AmmH model to combine the US and IRT images for the classification of thyroid nodules	2,864 patients (1,536 labeled as benign, and 1,328 labeled as malignant)	On the collected dataset, our AmmH model respectively achieved 97.17% and 97.38% of F1 and F2 scores, which significantly outperformed the single-modal models. The results of four ablation experiments further show the superiority of our proposed method.	The proposed multi-modal model extracts features from various modal images, thereby enhancing the comprehensiveness of thyroid nodules descriptions. The model has demonstrated promising classification performance, indicating its potential as a non-invasive, radiation-free, and cost-effective screening tool for distinguishing between benign and malignant thyroid nodules.
***Detection of nodules (regardless of their benignity)***				
Çetinkaya et al., 2018, Turkey	To investigate the efficacy of DITI, based on a noninvasive system, to display and detect the relative temperature variations in patients with thyroid nodules.	27 F (ages 29–68 years, mean 53.4years) and 5 M (ages 39–70 years, mean 55.2 years); 16 healthy volunteers, 12 F and 4 M served as control, with mean age 48.6 years and range 25–72 years	The thermal image shows the variation of the patient thyroid surface skin temperature according to hypoechoic and hyperechoic nodules. The DITIs showed higher temperature for hypo echoic nodule sides and it correlated with nodule size. There was no significant temperature difference on the sex distribution.	In conclusion, the present study suggests that an internal heat source is dependent of the noduls echogenity and size; ITI may be the first signal that such a possibility is developing. However, DITI is not a replacement for or alternative to US or any other form of thyroid nodules imaging. These are complementary procedures, one test does not replace the other.
Damião et al., 2020, Brazil	To evaluate thermographic images to analyze whether nodules can be identified in these images, considering the superficial skin temperatures measured by the infrared camera.	25 patients with thyroid nodules	Study 1 (Simulations): Fat tissue thickness significantly influences surface temperature; nodules are not always detectable in obese patients; Study 2 (CNN Results): Accuracy = 92%, Precision = 100%, Recall = 80%	IRT is promising for detecting thyroid nodules in non-obese individuals with thinner neck fat; CNNs can identify nodular regions with high precision, but model needs more training data; IRT is not suitable for patients with thick subcutaneous fat layers
de Souza et al., 2019, Brazil	To perform multi-modality imaging fusion.	A 40-year-old F with thyroid nodules and goiter diagnosis.	Higher temperature variations in the neck are found at the thyroid region, especially indicating nodules. The thyroid's surface temperature distribution aids in differentiating healthy from tumorous tissue. In this case study, MRI provides anatomical information, while infrared images offer functional insights into the thyroid gland.	This preliminary investigation provides proof of improvements in image registration for applications in medical diagnosis and for planning appropriate treatment.
Etehadtavakol et al., 2023, Iran, Singapore	To analyze thermographic images with the aim of enhancing interpretations of thyroid nodules through radiomics feature selection from thermal images of the thyroid gland.	53 patients with thyroid nodules	Radiomics analysis can be applied to thyroid thermography images to quantify temperature distribution, capture textural patterns, extract shape and size features, analyze heat patterns linked to blood flow, assess asymmetry between thyroid lobes, and monitor dynamic temperature changes over time.	Radiomics analysis holds significant potential to enhance the assessment of thermography thyroid images and provide valuable insights into thyroid function and pathology.
Etehadtavakol et al., 2024, Iran, Singapore	To explore the potential of AI, specifically CNNs, in enhancing the analysis of thyroid thermograms for the detection of nodules and abnormalities.	46 patients with thyroid nodules and abnormalities (5 studies: 46; 46; 92; 92; 46–92-92−46 images)	The best performance was in study 4 with k = 10, using 92 images, achieving an average accuracy of 0.9784, average loss of 0.0646, and average dice coefficient of 0.2857. For k = 6, the U-Net VGG16 model yielded the highest dice coefficient (0.2639), while ResNet50 and Inception-v3 had values of 0.1598 and 0.1844, respectively. ResNet50 achieved the highest accuracy (0.9775).	The findings here underscore the potential of AI for precise and efficient segmentation of thyroid nodules, paving the way for improved thyroid health assessment.
Flores et al., 2023, Mexico, Spain, USA	To identify hot areas in the neck regions, as a first approach for automatic classification of thermal images	31 nodules, with 6 thermographic images per nodule, of which 80.64% were F and 19.35% were M. The authors also used other databases	As can be seen the neural network can predict and segment the affected region in 84.21% of the cases	These findings suggest that the temperature change in thyroid nodules, compared to healthy thyroid tissue, is closely related to a higher risk of malignancy. ANNs have demonstrated their ability to accurately segment affected regions of the thyroid, showcasing their potential for applications in this domain.
González et al., 2017, Brazil	To propose a protocol for infrared image acquisition and a database for thyroid study with infrared images and clinical data and a method for ROI autonomous identification and image registration of thyroid region	25 volunteers (twenty with benign thyroid nodules, and 5 healthy as control group); The average age was 54 and F was the predominant gender	An algorithm based on rigid transformation to images registration is implemented and evaluated. Ninety-one percent (91%) of images were correctly registered.	A new database was created and published for researches on thyroid nodules diagnosis. They proposed an autonomous ROI identification method, which is based on very simple fundamentals of computers vision.
González et al., 2021, Brazil	To examine parameters affecting IR detectability of nodules	Simulated + real data; number not specified	Depth and tissue thickness affect surface temperature	The analysis of the patient's infrared examinations supports all findings. It shows that fat layer thickness significantly impacts thermal insulation. Differences in vascular patterns of nodules, despite similar diagnostics, dimensions, and US characteristics, do not affect surface temperature, confirming the results of the numerical study.
Moran et al., 2018, Brazil	To find regions of thyroid nodules in general in order to segment nodular regions in thermograms to facilitate the M.D. examination	92 images of thyroid regions: 39 nodule related and 53 healthy regions.	The GoogLeNet CNN yielded the highest accuracy (86.22%) followed by AlexNet (77.67%) and the VGG (74.96%).	The major contributions of this work were: (1) the development of a novelty methodology for thyroid nodule disidentification thermograms and (2) the demonstration of the feasibility of the use of CNNs in thyroid nodules identification.
Vardasca et al., 2019, Portugal	To investigate the thermal pattern of a patient presenting with a hypervascular thyroid nodule	A 40-year-old M with an 11 x 6 mm left thyroid nodule	The ROI presented at baseline a bilateral difference in mean temperature of 0.4ºC, after cooling this difference was accentuated, the affected side recovered quickly and showed a hotspot in the area of the nodule identified by Doppler imaging.	This case study showed evidence of the utility on using dynamic infrared thermal imaging when assessing thyroid nodules, which was confirmed by Doppler imaging to be highly vascularized and presenting a higher skin temperature when compared with the contralateral side. For diagnostic purposes, the traditional expensive methods such as biopsy and nuclear medicine are still required.
Recurrence				
Camargo et al., 2019, Brazil	To analyze the viability of thermal imaging in the evaluation of thyroid cancer recurrence.	A 34-year-old F patient participated in the study. She underwent right lobectomy of the thyroid gland with diagnosis of nodular neoplasm adenoma in the upper third of the left lobe of the thyroid	The average of temperature difference, compared to the healthy tissue (isthmus) after cold stressing is 1.0º C after 5 or 10 minutes and 0.8ºC after 15 minutes. The temperature ripple was higher on the healthy tissue (5°C) than on the cancerous one (3.6ºC) right after cold stressing.	IRT seems to be effective in detecting the new thyroid nodule in the left lobe.

*ΔT* temperature delta; *AI* artificial intelligence; *AmmH* adaptive multi-modal hybrid; *ANN* artificial neural network; *AUC* area under the curve; *CNN* convolutional neural network; *CT* computed tomography; *DITI* digital infrared thermal imaging; *DIRT* dynamic infrared thermography; *F* female; *Ft* final temperature; *HC* healthy controls; *IR* infrared; *IRT* infrared thermography; *It* initial temperature; *LASSO* least absolute shrinkage and selection operator; *M* male; *M.D.* medical degree; *Mt* medium temperature; *MRI* magnetic resonance imaging; *P*_*A*_ asymmetry parameter; *ROC* receiver operating characteristic; *ROI* region of interest; *TA* thyroid adenoma; *TC* thyroid carcinoma; *TN* thyroid nodules; *US* ultrasound

Regarding type of IRT, of the studies that reported the type of IRT, 14 studies used SIRT [7, 11, 12, 15, 16, 18, 20, 24, 27–29, 31–33] (Table 2). In all studies with DIRT, cold stress was used as thermal stimulus (Table 3). Many of the included studies used AI classifiers to aid in the diagnosis of thyroid nodules [5, 11, 12, 15, 17, 20, 22–25, 31, 32]. Other studies used software and libraries for preprocessing and processing of images (Table 4) [7, 21, 26, 27, 30, 34, 35].

**Table 2:** Summary of results: thermal camera, and Static IRT imaging*

Author/year	Thermal camera used	IRT used
Çetinkaya et al., 2018, Turkey	FLIR E8, 320 × 240 pixels	Static
D’Arbo Alves et al., 2016, Brazil	NA	Static
Damião et al., 2020, Brazil	FLIR T620sc, 640 × 480 pixels	Static
de Souza et al., 2019, Brazil	FLIR A325, 320 × 240 pixels, temperature range from − 20 to 120 °C, and 0.08 °C in thermal resolution	Static
Etehadtavakol et al., 2024, Iran, Singapore	NA	Images from 46 patients were selected available in the dataset
Flores et al., 2023, Mexico, Spain, USA	FLIR T-600, 640 × 480 pixels, sensitivity of less than or equal to 0.04 °C.	Static
González et al., 2021, Mexico	FLIR SC620, sensitivity of less than 0.040 °C, 640 × 480 pixels)	Static
Gavriloaia et al., 2009, Romania	10 Megapixel Digital Camera with microbolometers, 240 × 320 pixels, temperature resolution of 0.10 °C	Static
Gavriloaia et al., 2009, Romania	Resolution of 320 × 240 pixels, the thermal resolution of 0.10 °C	Static
Moran et al., 2019, Brazil	FLIR SC 620, 640 × 480 pixels	Static
Pathak et al., 2022, India, United Arab Emirates	FLIR SC-620, 1,280 × 720 pixels, temperature range − 40 °C to 40 °C	Static
Perpetuini et al., 2023, Italy	FLIR SC660, 640 × 480 pixels	Static
Zhang et al., 2023, China	HB-T-1 Thermal Imaging System, 320 × 240 pixels, temperature resolution of 0.08 °C	Static

*NA* not available;

* The study by González et al. (2019) employed both static and dynamic IRT using a FLIR SC620 camera with a resolution of 640 × 480 pixels

**Table 3:** Summary of results: thermal camera, and dynamic IRT imaging

Author/year	Thermal camera used	IRT used
Bahramian and Mojra, 2020, Iran	FLIR A655sc, 640 × 480 pixels; thermal sensitivity less than 0.03 °C (30 mK) at 30 °C)	Dynamic (the neck skin was cooled with a spray of medical ethanol solution (25% water and 75% medical ethanol).
Camargo et al., 2019, Brazil	Fluke TI 32, 320 × 240 pixels, thermal sensitivity lesser than or equal to 0.045 °C	Dynamic (cold stressing was performed, with a gel bag cooled at 8 °C throughout the neck, holding 30 s.)
Camargo et al., 2022, Brazil	Fluke TI 32, 320 × 240 pixels, spatial resolution of 0.63 mrads, thermal sensitivity less than or equal to 0.045 °C	Dynamic (cold stress)
Chantasartrassamee et al., 2024, Thailand	tcam-mini thermal camera connected to an iPad mini, 320 × 240 pixels, operating in a temperature range of 25–40 °C	Dynamic (a cooling gel (estimate to be at 8 °c) was applied to the neck region for 30 s)
Damião et al., 2021, Brazil	FLIR SC620, 640 × 480 pixels, sensitivity < 0.04 °C	Dynamic (The patient’s neck area was cooled evenly with a flow of air from a ventilator until the temperature indicated by the toolbox reached 31.5 °C. The camera was configured to capture an image every 15 s for 5 min, resulting in a total of 20 images.
Etehadtavakol et al., 2023, Iran, Singapore	FLIR SC 620, 640 × 480 pixels, sensitivity < 0.04 °C	Dynamic (The patient’s neck area was uniformly cooled using airflow from a ventilator until the temperature indicated by the toolbox reached 31.5 °C.)
González et al., 2017, Brazil	FLIR SC620, 640 × 480 pixels, sensitivity smaller than 0.040 °C	Dynamic (An air flow (electric fan) was directed to the patient. One image was captured every 15 s over 5 min, producing one sequence of 20 images)
González et al., 2019, Brazil	FLIR SC620, 640 × 480 pixels	Static and dynamic (used cooling via airflow, 20 frames over 5 min)
González et al., 2021, Brazil	FLIR SC620, 640 × 480 pixels.	Dynamic (neck cooling by an airflow (made by an electric fan) until it reaches a mean value of 30 C or for 5 min, as the maximum cooling time)
Gavriloaia et al., 2010, Romania	NA	NA
Salles et al., 2020, Brazil	Fluke TI32, thermal sensitivity ≤ 0.045 °C, 320 × 240 pixels	Dynamic (cold stress was performed, with a gel bag cooled to 8 °C over the entire length of the neck, being maintained for 30 s).
*Schadeck* et al., 2020, Brazil	Fluke Ti4000, 320 × 240 pixels	Dynamic (cold stress, using cooled gel bags applied tothe region of interest for two minutes in contact with the skin surface).
Vardasca et al., 2019, Portugal	FLIR E60, 320 × 240 pixels	Dynamic (The aluminum disks were applied by conduction for 1 min on both sides of the anterior neck and above the laryngeal prominence, 5 images were taken at 1-minute interval.

*NA* not available

* The study by González et al. (2019) employed both static and dynamic IRT using a FLIR SC620 camera with a resolution of 640 × 480 pixels

**Table 4:** Summary of the included studies that used artificial intelligence tools

Author/year	Algorithms/computer systems or image processing method used	ROI selection
Bahramian and Mojra, 2020, Iran	Bayesian segmentation; Perona-Malik anisotropic diffusion filter; MATLAB (R2016a); Edge detection + 2D/3D visualization	Based on anatomical landmarks and temperature rise near thyroid; ROI box over neck; max temperature used to locate hotspot
Camargo et al., 2022, Brazil	Dynamic (gel pack cooled to 8 °C, being placed across the neck, for 30 s.)	Volunteers were positioned lying down with cervical extension, supported by a cushion. Metallic markers were placed on the mentonian protuberance and sternal notch to define the analysis region.
Chantasartrassamee et al., 2024, Thailand	ResNet50V2 (deep CNN)	An initial image was captured before cooling, followed by images every 15 s for 5 min, totaling 22 images per participant.
Damião et al., 2020, Brazil	ResNet CNNs were used in the classification process	Automatic segmentation based on thermal asymmetry, followed by morphological operations (opening, erosion, dilation) to refine ROIs, then cropping and resizing for CNN input.
de Souza et al., 2019, Brazil	Based on the Moore-Neighbor Tracing algorithm with Jacob’s stopping criterion; Non-rigid geometric image registration using B-splines; implemented in MATLAB;	Manual segmentation and thresholding of IR images; registration points selected semi-automatically from common anatomical locations (eye, ear, mouth)
Etehadtavakol et al., 2023, Iran, Singapore	PCA, ICA, and variance thresholding were employed to reduce the dimensionality of the feature set	The dataset included 53 annotated images, some with multiple regions of interest. To address the data insufficiency, each region was cropped individually, resulting in 75 distinct labeled images for feature extraction.
Etehadtavakol et al., 2024, Iran, Singapura	A fusion of U-Net and VGG16, combined with feature FE	Images were preprocessed for AI segmentation by cropping and resizing rectangular images (640 × 480) to 480 × 480. For studies 1–3, images were resized to 240 × 240, and for study 5, to 256 × 256 with normalization.
Etehadtavakol et al., 2025, Iran, Singapore, USA	A set of machine learning models, including SVM, Random Forest, Decision Tree, AdaBoost, and XGBoost, were tested as classifiers through group k-fold cross-validation	ROIs were automatically extracted from binary masks using thresholding, morphological closing, and removal of regions smaller than 200 pixels.
Flores et al., 2023, Mexico, Spain, USA	An ANN based on the Nested U-Net architecture is trained and used to detect thyroid pathologies	The Nested U-Net has encoder-decoder subnetworks connected by nested paths, with a Softmax output. The error is calculated using BCE and Dice Loss, optimized with Adam, 1500 epochs, and a batch size of 6.
González et al., 2017, Brazil	k-NN	Automatic detection of neck and sub-neck region based on anatomical geometry; validated by specialists
González et al., 2019, Brazil	ROI-based temperature extraction; time-series clustering via k-Means; histogram-based thresholding; segmentation via prior algorithms	ROI from neck and upper chest, subdivided into 11 × 11 px squares; insulation squares used to highlight nodules; analysis focused on temperature ranges and series
González et al., 2021, Brazil	COMSOL simulation (for bioheat model)	After thermal stress, 20 infrared images are captured every 15 s for 5 min. An additional image marks nodule positions using insulating thermal material, applied by a medical doctor after nodule identification.
González et al., 2021, Mexico	Amazon SageMaker ground truth portal	The acquired images were postprocessed by manually segmenting them into six regions. The values obtained for each region were the mean, maximum, and minimum temperatures.
Gavriloaia et al., 2009, Romania	Adaptive spline interpolation; computer fusion software integrating video + thermistor readings	Rectangular area over thyroid selected manually; analyzed for asymmetry, irregular contour, and elevated temperature
Gavriloaia et al., 2010, Romania	Fractal analysis was applied to each ROI: the fractal dimension was calculated using the box-counting method, while lacunarity was determined through the gliding box algorithm, based on mass distribution analysis.	Thermal images were segmented into individual ROIs for analysis. Segmentation was performed either automatically, where the software identified the largest and darkest object in the image, or manually, where the user selected the area of interest in cases with multiple or overlapping objects.
Moran et al., 2018, Brazil	The CNNs used were based in GoogLeNet, AlexNet and VGG architectures	Semi-automated: based on symmetry-based thermal asymmetry (> 0.3 °C); morphological erosion used to refine potential nodule regions
Moran et al., 2019, Brazil	CNN algorithms based on the GoogLeNet and ResNet architectures.	- Initial segmentation based on P_A_> 0.3 °C) - Region refinement using grayscale-level grouping and morphological operations
Pathak et al., 2022, India, United Arab Emirates	CNN for classification	Thermal image handling includes capture, preprocessing, data augmentation, segmentation, classification, and analysis. Initial images undergo augmentation, followed by segmentation. The model is trained, tested, and refined based on outcomes.
Salles et al., 2020, Brazil	Python software using FloodFill algorithm for region segmentation; image analysis via exported CSV files	User clicks seed point in nodule; algorithm expands region using temperature threshold (semi-automatic); tracks ROIs across frames using interpolation
*Schadeck* et al., 2020, Brazil	Python (v3.8), Region Growing Algorithm, NumPy, PIL, Tkinter; GUI interface for analysis	Semi-automatic via user-selected seed pixel; segmentation based on thermal similarity (threshold criterion); compared to manual ROI in SmartView
Zhang et al., 2023, China	AmmH, VGG16, Inception V3, ResNet18, and ViT.	The examiner acquired the IRT image by placing the device’s probe on the subject’s neck, ensuring the neck is centered, with the upper screenshot at ear level and the lower at shoulder level.

*AI* artificial intelligence; *ANN* artificial neural network; *BCE* binary cross entropy; *CSV* comma separated volumes; *FE* feature engineering; *ICA* independent component analysis; *IR* infrared; *IRT* infrared thermography; *k-NN* k-Nearest Neighbors; *MATLAB* Matrix laboratory; *P*_*A*_asymmetry parameter; *PCA* principal component analysis; *ROI* region of interest; *SVM* support vector machine; *VGG* visual geometry group

### Results of individual studies

#### Detection and differentiation of benign and malignant nodules

One of the studies compared only benign and malignant nodules [19]. This study aimed to evaluate the temperature differences between benign and malignant thyroid nodules by using IRT in 113 patients (27 malignant and 120 benign nodules); ΔT (2.95 °C vs. 1.73 °C, *p* < 0.0001) was higher in the malignant nodule group than in the benign nodule group. Other studies compared IRT in healthy patients and in patients with nodules [8, 28, 33]. Gavriolia et al. [28] investigated IRT signatures of thyroid nodules of 24 patients. The authors observed that malignant tumors have a distinct infrared signature. Only the area affected is thermally registered, which is characterized by irregular shape and strong non-uniform structure with rapid variations in skin temperature [28].

De Camargo et al. [8] analyzed the thermal behavior of thyroid nodules (42 nodules and 9 controls) using cold stress. Thermal differences between control and study groups (*p* < 0.001) were revealed. Furthermore, the authors concluded that benign nodules present a different thermal behavior than malignant nodules, and both present different behavior than normal tissue. Similarly, Perpetuini et al. [33] examined the capability of IRT applied to the neck skin to distinguish thyroid carcinoma, thyroid adenoma, and healthy controls without the use of cold-stress protocols. Significant differences were observed in both maximum (*p* = 0.023) and average temperatures (*p* = 0.002). However, statistical differences for average temperature (*p* = 0.027) were only noted between thyroid carcinoma and healthy controls. In contrast, the maximum temperature showed differences between thyroid carcinoma and both thyroid adenoma (*p* = 0.021) and healthy controls (*p* = 0.002).

Two of the included studies evaluated the applicability of IRT with subsequent US confirmation [16, 36]. Centikaya et al. [16] analyzed the efficacy of IRT to display and detect the relative temperature variations in thyroid surface skin (32 nodules and 16 healthy volunteers) and found variations in thermal images according to the nodule echogenicity. The IRT showed higher temperature for hypoechoic nodules and it correlated with nodule size. The authors concluded that IRT is a valuable auxiliary to US, especially in thyroid hypoechoic nodules. On the other hand, Vardasca et al. [36] reported the case of a 40-year-old male patient (with a 11 × 6 mm nodule at the left side of the thyroid gland), and showed evidence of the utility of using DIRT when assessing thyroid nodules, which was confirmed by Doppler imaging to be highly vascularized and presenting a higher skin temperature when compared with the contralateral side.

In their study, D’Arbo et al. [18] conducted a comparative analysis of power Doppler US and IRT to evaluate their effectiveness in selecting thyroid nodules for FNA. The findings revealed that, relative to FNA results, power Doppler US exhibited a sensitivity of 95.16%, a specificity of 23.52%, a positive predictive value (PPV) of 96.22%, a negative predictive value (NPV) of 16.70%, and an overall accuracy of 89.51%. Conversely, IRT demonstrated a sensitivity of 100%, specificity of 58.06%, PPV of 87.73%, NPV of 100%, and an identical accuracy of 89.51%. Based on these metrics, the authors concluded that IRT provided greater precision than power Doppler US in identifying thyroid nodules warranting biopsy.

#### Hybrid models for detecting thyroid nodules

de Souza et al. [20] presented the combination of MRI with IRT images (representing functional information) applied to a case study of a 40-year-old female patient diagnosed with thyroid nodules and goiter. This combination proved effective for both anatomical information (obtained through MRI) and functional information (obtained through IRT). Nevertheless, it is important to note that this was a single case study.

Zhang et al. [11] used multi-modal learning by fusing IRT and US, where the model demonstrated promising classification performance, indicating its potential as a non-invasive, radiation-free, and cost-effective screening tool for distinguishing between benign and malignant thyroid nodules. The fusion of multi-modal data has been a research focus in the field of multi-modal learning, as it allows models to benefit from different data modalities by learning complementary information [11].

#### IRT as an auxiliary tool in the detection of cancer recurrence

One of the included studies evaluated the feasibility of IRT in the evaluation of thyroid cancer recurrence in a patient who underwent right lobectomy of the thyroid gland with diagnosis of nodular neoplasm adenoma in the upper third of the left lobe of the thyroid [14]. The temperature ripple was higher in the healthy tissue (5 °C) than in the cancerous tissue (3.6 °C) immediately after cold stressing. The authors concluded that IRT appears to be effective in detecting new thyroid nodules.

#### AI classifiers to aid in the diagnosis of thyroid abnormalities

Eleven studies investigated AI classifiers in the detection of thyroid abnormalities [5, 11, 12, 15, 17, 22–25, 31, 32]. In the study by Chantasartrassamee et al. [17], the pre-trained ResNet50v2 model was used as a deep learning algorithm for infrared thermal image recognition to differentiate between benign and malignant thyroid nodules. Before skin cooling (application of an 8 °C colling gel for 30 s), the model achieved an accuracy of 0.75, and a sensitivity of 0.78. The machine learning model’s accuracy during the post-cooling phase was 0.69, but the difference was not statistically significant compared with the pre-cooling phase (*p* = 0.236). Furthermore, the AI model that integrated preoperative thyroid US findings and cytological results demonstrated slightly higher results compared to the AI model evaluated during the pre-cooling phase, yielding an accuracy of 0.81 and a sensitivity of 0.84.

Flores et al. [24] evaluated an ANN based on the Nested U-Net architecture to detect thyroid nodules, and the neural network could predict and segment the affected region in 84.21% of the cases. Results showed that when calculating the correlation coefficient between the average temperature gradient (∇T) and the results of thyroid US, expressed in the American College of Radiology TI-RADS classification, a high degree of correlation was obtained. The corrected η2 was 0.913 (*p* < 0.001), and a 95% confidence interval of 0.809 to 0.936, obtained through one-way ANOVA for the average ∇T.

In the study by Damião et al. [15], ResNets were used in the classification process for thyroid nodule identification. The training process achieved accuracy of 96% for in-sample and 95% for validation. In the testing phase, 92% accuracy, 100% precision and 80% recall were achieved. In the study by Etehadtavakol et al. [22], a hybrid model combining U-Net and VGG16 with feature engineering was proposed for thyroid nodule and abnormality detection. The highest Dice coefficient (k = 6) was achieved by the U-Net–VGG16 model (0.2639), compared with 0.1598 for ResNet50 (feature engineering) and 0.1844 for Inception-v3 (feature engineering). However, the highest accuracy was obtained with ResNet50 (0.9775).

A semi-automatic method for image registration was implemented to pre-process the thermograms, and a new method for ROI extraction was proposed in the study by González et al. [25]. Furthermore, the k-Nearest Neighbours (k-NN) algorithm was used to perform classification using the time series from extracted features. In this study, 91% of images were correctly registered. In the study by González et al. [12], using ROI-based temperature extraction, time-series clustering via k-Means, histogram-based thresholding, and segmentation via prior algorithms, it was observed that no consistent temperature threshold differentiated benign vs. malignant nodules, malignant regions were not always the hottest part of ROI, and cluster analysis (k-means) did not clearly segment nodule regions unless k ≥ 5.

In the study by Moran et al. [5], three convolutional neural networks (CNN) (GoogleNet, AlexNet, and VGG architecture) were tested to identify thyroid nodules in thermograms by using simple image processing techniques. The GoogLeNet CNN yielded the highest accuracy (86.22%), followed by AlexNet (77.67%) and VGG (74.96%). Moran et al. [31] also investigated CNN algorithms, this time based on the GoogLeNet and ResNet architectures. Three learning rate values and five datasets were tested to maximize the performance of the algorithms. The ResNet CNN using the DT4 dataset as a training set led to the best results in their experiments (accuracy = 0.9584). Similarly, Pathak et al. [29] used CNN to detect malignant nodules from thermograms for hyperthyroidism, and the trained CNN had a classification accuracy of 91.11%, a precision of 0.9167, and a recall value of 0.93076.

In the study by Zhang et al. [11], a novel Adaptive multi-modal Hybrid (AmmH) model was tested to combine US and IRT images for the classification of thyroid nodules. The model achieved F1 and F2 scores of 97.17% and 97.38%, respectively, significantly outperforming single-modal models (VGG16, Inception V3, ResNet18, and ViT).

In the study by Etehadtavakol et al. [23], machine learning models (SVM, Random Forest, Decision Tree, AdaBoost, and XGBoost) were evaluated as classifiers using group k-fold cross-validation. The achieved accuracies were 83% for Random Forest, 79% for AdaBoost, 78% for Decision Tree, and 82% for XGBoost. Among the feature selection methods, LASSO (a supervised embedding-based feature selection approach) demonstrated the best performance, reaching 86% accuracy with an AUC of 0.91 for the Random Forest model and 86% accuracy with an AUC of 0.92 for the XGBoost model.

#### Software and libraries for the preprocessing and processing of images

Some of the included studies used software and libraries for preprocessing and processing of IRT [7, 20, 21, 26, 27, 29, 30, 34, 35]. Bahramian and Mojra [7] aimed to analyze the thermograms regarding the parameters of malignant thyroid tumors, using Bayesian segmentation, Perona-Malik anisotropic diffusion filter, MATLAB (R2016a), and Edge detection with 2D/3D visualization. The authors concluded that IRT could localize and estimate the shape of angiogenesis of malignant thyroid tumors and affirmed the strong potential for early cancer diagnostics and numerical modelling.

In the study by de Souza et al. [20], a combination of MRI and thermograms was applied to a case study of the thyroid gland. The point selection was based on the Moore-Neighbour Tracing algorithm with Jacob’s stopping criterion, and Non-rigid geometric image registration using B-splines was implemented in MATLAB. The MRI delivers detailed anatomical and structural information about the thyroid gland, while functional data is derived from infrared imaging. However, it is important to note that although MRI images offer high spatial resolution, infrared images are significantly limited in this regard.

Etehadtavakol et al. [21] employed a machine learning technique to analyze thermograms, aiming to enhance the interpretation of thyroid nodules by selecting radiomics features from thermograms of the thyroid gland. In this study, principal component analysis, independent component analysis, and variance thresholding were employed to reduce the dimensionality of the feature set. The authors concluded that radiomics analysis, which focuses on extracting large amounts of quantitative data from medical images, holds significant potential to enhance the assessment of thermograms and provide valuable insights into thyroid function and pathology.

In the study by González et al. [26], a simulated bioheat model created using COMSOL, together with real data, was used to examine parameters affecting IRT detectability of nodules. The authors found that differences in vascular patterns of nodules (with the same diagnosis, similar dimensions, and ultrasonographic characteristics) have no influence on the temperature of the surface during the examination of real patients, thus confirming the results of the numerical study. González et al. [27] developed a thermal interpretation procedure that could eventually become a standard for detecting thermal anomalies in the neck, which may indicate thyroid pathologies. In this study, the authors utilized the Amazon SageMakerGroundTruth portal for manual image segmentation. From the thermal patterns analyzed in this study, it was concluded that values of temperature difference ≥ 0.5^°^C are consistent with thyroid pathology, and values of temperature difference ≥ 1.0 °C are more indicative of malignant thyroid pathology.

Gavriloaia et al. [29] developed and assessed a high spatial-temperature resolution mapping system for early cancer diagnosis, including thyroid cancer. The system used adaptive spline interpolation and computer fusion software that integrated video data with thermistor readings. In this study, the authors observed that cancerous ROIs were more asymmetrical and ~ 1.5 °C hotter than surrounding tissue. Furthermore, non-cancerous ROI showed a ~ 0.5 °C difference, and the thermistor offered superior temperature resolution (0.04 °C) vs. infrared camera.

In the study by Gavriloaia et al. [30], the segmentation of regions of interest was carried out either automatically, where the software detected the largest and darkest object in the image, or manually. Subsequently, fractal analysis was applied to each ROI: the fractal dimension was computed using the box-counting method, while lacunarity was assessed with the gliding box algorithm based on mass distribution analysis. In this study, the fractal dimension was 1,4217 for hyperthyroidism and 1,818 for papillary cancer.

Salles et al. [34] developed and presented a semiautomatic segmentation software. In this study, Python software with the FloodFill algorithm was used for region segmentation. The software allowed detailed tracking of temperature variation and region size over time. Schadeck et al. [35] developed a semiautomatic segmentation model of thermograms using the Python computational language. The developed software was able to segment the ROI of breast and thyroid thermograms. In addition, the proposed model comprises the tumor region with greater reliability than the manual ROI delimitation method.

#### Limitations in the detection of thyroid nodules

In the bioheat transfer analysis conducted by Damião et al. [15], different fat thicknesses were considered, as the surface temperature of the neck may be related to the thickness of the fat layer due to the insulating effect of adipose tissue. The authors performed simulations of malignant nodules in necks with fat tissue layer thicknesses of 1.2 cm, 0.6 cm, and 0.1 cm. The authors concluded that patients with a considerable fat layer in the neck area are not candidates for nodular detection by IRT examinations: such an examination is not indicated for obese or even overweight individuals.

In line with this, the study by González et al. [26] with bioheat transfer analysis demonstrated a significant thermal insulation effect of the fat tissue layer. This indicates that the impact of heat generated by a thyroid nodule on the skin’s surface will diminish if the fat tissue layer in that area is thick. Additionally, both during transient and steady states, the lowest temperature variations were recorded on the skin surface when the thicker fat layer was present, showing that this layer reduces the effect of the nodule on the neck’s surface.

## Discussion

This scoping review examined the applicability of IRT in detecting and classifying thyroid nodules. A total of 28 studies were identified, employing both SIRT and DIRT, using cameras with varying resolutions, as well as different preprocessing and image processing tools. Additionally, AI techniques were applied to assist in the detection of thyroid nodules. Among the studies included in this review, 14 employed SIRT. This technique captures the thermal distribution at a single point in time, making it susceptible to errors and influenced by individual variability, external measurement conditions, and technical limitations. Moreover, SIRT does not allow for the assessment of responses to external stimuli. While this limitation could be mitigated by periodically capturing thermal images, DIRT inherently provides continuous temperature measurement without the need for repeated image acquisition [37].

Some studies observed distinct thermal behavior following thermal stress application and analysis using DIRT. The thermal stress protocol used by Camargo et al. [8] demonstrated that benign and malignant nodules exhibit significantly different thermal parameters (*p* < 0.001), which also differ from those of normal tissue. In the study by Salles et al. [34], dynamic segmentation software detected larger and hotter regions in malignant nodules compared to benign ones. In the case study by Vardasca et al. [36], a hypervascularized nodule (confirmed by Doppler imaging) showed accelerated thermal recovery and a visible hotspot after cooling. Bahramian and Mojra [7] observed that malignant nodules exhibited a local temperature increase of 1–1.5 °C compared to healthy tissue. Moreover, Damião et al. [19] reported that malignant nodules exhibit higher temperatures than benign ones in thermographic evaluations.

Although the studies indicate a tendency for malignant lesions to exhibit higher temperatures compared to benign nodules and healthy tissue, it is essential to consider the lack of standardization in acquisition protocols, as well as the heterogeneity of materials and resources used to induce thermal stress. These factors may contribute to increased variability in the results.

Most of the cameras used in the studies had a moderate resolution (ranging from 320 × 240 pixels to 640 × 480 pixels). One study, however, used a new high-resolution camera model available on the market (1280 × 720 pixels) [32]. The detail a thermal camera can detect depends on its resolution. Thermal cameras with high resolution offer greater precision in detecting temperature variations, which significantly improves diagnostic accuracy. In fields where precision is vital, such as medical diagnostics, this capability can substantially enhance overall performance.

To further improve accuracy, some studies have employed hybrid models for detecting thyroid nodules [11, 20]. Medical imaging technologies are classified into two main categories: anatomical and functional. To overcome the limitations of information provided by a single imaging modality, image registration and fusion techniques are being applied to generate blended images, enabling better interpretation and clinical diagnosis. However, image registration and fusion are not trivial tasks, mainly due to differences in the capture conditions of the modalities involved [20].

In addition to hybrid models, other studies included in this review used AI tools, as well as automatic and semi-automatic segmentation techniques, to enhance the visualization of thermal images and improve the final diagnosis. AI techniques are highly effective in identifying and diagnosing different types of diseases. The use of computer-based reasoning as a method to enhance medical services offers unprecedented opportunities to improve patient outcomes and reduce healthcare service costs [38].

In this context, most of the included studies used various types of ANN, primarily CNNs [5, 11, 15, 17, 22, 24, 31, 32], while only two studies used simple machine learning algorithms exclusively [12, 25]. One of the studies used k-NN [27], while the other used k-means clustering [12]. Conventional ANNs consist of interconnected nodes, or “neurons,” each performing a simple operation; collectively, these layers transform input data into increasingly abstract representations, ultimately producing an inference. Pre-trained models further enhance this process by leveraging features learned from large benchmark datasets, allowing them to be adapted to medical imaging tasks with relatively little additional training [39].

Among the included studies, there was variability in the accuracy of the proposed ANN models. While the study by Chantasartrassamee et al. [17] reported an accuracy of 75% for detecting malignant nodules, Damião et al. [15] showed that ANN models achieved 92% accuracy in detecting thyroid nodules. Regarding hyperthyroidism cases, excellent accuracy results were observed. Pathak et al. [32] achieved an accuracy of 91.11% when using CNNs to detect cancerous nodules from thermograms related to hyperthyroidism.

CNNs have been widely used for feature extraction from input data, including medical images, and have demonstrated remarkable proficiency in this task. However, they may struggle to capture long-range dependencies between features, which can limit their ability to fully represent complex data. In this context, studies such as Zhang et al. [11] proposed a novel AmmH model, which was tested to combine ultrasound and IRT images for the classification of thyroid nodules, achieving an F1 score of 97.17%.

Despite its broad applicability and several advantages, IRT has some limitations. Its main limitation is the sensitivity to external factors, such as ambient temperature and humidity, as well as the patient’s physical condition. Although factors such as ambient temperature and humidity can be controlled, another limitation is that fat thickness, particularly in obese patients [15], as well as the depth and thickness of tissues, can affect the thermal detection of nodules [26].

González et al. [12] concluded that in SIRT, similar temperature values were present on the surface of the neck skin for both benign and malignant nodules. In DIRT, it was observed that similar temperatures were found in nodular and healthy regions. The authors also suggested that this might be related to differences in the anatomical and biophysical parameters of the patients, which should also be analyzed and considered in the application of IRT.

### Strengths and limitations

To date, this is the first scoping review examining the role of IRT in the detection of thyroid nodules. Moreover, this study strictly adhered to the JBI Scoping Review Methodology Group protocol, which included multiple sources, enabling a high sensitivity in the literature search to identify the necessary studies that answered the established question and, consequently, achieved the proposed objective.

Some limitations of this study include the reliance on Google Scholar as the sole source of grey literature, as well as the inclusion of conference proceedings, which was necessary because research in computer science, particularly in AI, is often published in conference proceedings rather than journals. Additionally, methodological heterogeneity in primary studies limits ability to draw consistent and reliable conclusions across the evidence base.

### Implications for clinical practice and future research

Based on the review conducted, there is emerging evidence supporting the future implementation of IRT as an auxiliary tool in the diagnosis of thyroid nodules. In addition to being non-invasive, portable, non-ionizing, and contact-free, with significant advances in camera technology, the integration of AI has further enhanced the appeal of IRT in recent years. IRT emerges as a viable diagnostic method, especially in light of the limitations of current conventional techniques. Although the search yielded a considerable number of studies, there is noticeable variability in the protocols used, particularly in DIRT. Therefore, well-designed studies applying international standards and protocols for thermographic procedures are necessary to achieve more consistent results. Furthermore, considering certain limitations of IRT, such as reduced accuracy in the presence of increased adipose tissue or with deeper lesions, future studies should also investigate cases involving overweight or obese patients, as well as deeper lesions, to draw more robust conclusions.

## Conclusion

The use of IRT as a tool for thyroid nodule assessment is still emerging. Although it is not yet a widely adopted diagnostic method, IRT, particularly with AI support, offers promising potential for early, non-invasive thyroid nodule screening. Further research involving modern infrared thermal cameras and larger samples exploring IRT detection of thyroid nodules with AI is needed to fully evaluate the capabilities of this imaging modality.

**Note.** ΔT, temperature delta; AI, artificial intelligence; AmmH; adaptive multi-modal hybrid; ANN, artificial neural network; AUC, area under the curve; CNN, convolutional neural network; CT, computed tomography; DITI, digital infrared thermal imaging; DIRT, dynamic infrared thermography; F, female; Ft, final temperature; HC, healthy controls; IR, infrared; IRT, infrared thermography; It, initial temperature; LASSO, least absolute shrinkage and selection operator; M, male; M.D., medical degree; Mt, medium temperature; MRI, magnetic resonance imaging; P_A_, asymmetry parameter; ROC, receiver operating characteristic; ROI, region of interest; TA, thyroid adenoma; TC, thyroid carcinoma; TN, thyroid nodules; US, ultrasound;

## Electronic Supplementary Material

Below is the link to the electronic supplementary material.

Supplementary Material 1
