# Supplementary material for: The role of infrared thermography in thyroid nodule detection and classification: a scoping review
Source: Eur Arch Otorhinolaryngol. 2026 Jun 1;283(9):5421–38. doi: 10.1007/s00405-026-10309-6 (PMC13615063; doi:10.1007/s00405-026-10309-6)
Supplement: Supplementary file 1 [file 405_2026_10309_MOESM1_ESM.docx]

### **Appendix I: Search strategy**

| Database | Search Strategy (September 4, 2025) |
| --- | --- |
| PUBMED | (("infrared"[All Fields] AND ("thermographies"[All Fields] OR "thermography"[MeSH Terms] OR "thermography"[All Fields])) OR ("thermographies"[All Fields] OR "thermography"[MeSH Terms] OR "thermography"[All Fields]) OR (("thermal"[All Fields] OR "thermalization"[All Fields] OR "thermalize"[All Fields] OR "thermalized"[All Fields] OR "thermalizes"[All Fields] OR "thermalizing"[All Fields] OR "thermally"[All Fields] OR "thermals"[All Fields]) AND ("image"[All Fields] OR "image s"[All Fields] OR "imaged"[All Fields] OR "imager"[All Fields] OR "imager s"[All Fields] OR "imagers"[All Fields] OR "images"[All Fields] OR "imaging"[All Fields] OR "imaging s"[All Fields] OR "imagings"[All Fields])) OR ("thermography"[MeSH Terms] OR "thermography"[All Fields] OR "thermogram"[All Fields] OR "thermograms"[All Fields]) OR "therm*"[All Fields] OR ("infrared"[All Fields] AND "therm*"[All Fields]) OR ("thermography"[MeSH Terms] OR "thermography"[All Fields] OR ("temperature"[All Fields] AND "mapping"[All Fields]) OR "temperature mapping"[All Fields])) AND ((("thyroid gland"[MeSH Terms] OR ("thyroid"[All Fields] AND "gland"[All Fields]) OR "thyroid gland"[All Fields] OR "thyroid"[All Fields] OR "thyroid usp"[MeSH Terms] OR ("thyroid"[All Fields] AND "usp"[All Fields]) OR "thyroid usp"[All Fields] OR "thyroids"[All Fields] OR "thyroid s"[All Fields] OR "thyroidal"[All Fields] OR "thyroideal"[All Fields] OR "thyroidism"[All Fields] OR "thyroiditis"[MeSH Terms] OR "thyroiditis"[All Fields] OR "thyroiditides"[All Fields]) AND "nodule*"[All Fields]) OR (("thyroid gland"[MeSH Terms] OR ("thyroid"[All Fields] AND "gland"[All Fields]) OR "thyroid gland"[All Fields] OR "thyroid"[All Fields] OR "thyroid usp"[MeSH Terms] OR ("thyroid"[All Fields] AND "usp"[All Fields]) OR "thyroid usp"[All Fields] OR"thyroids"[All Fields] OR "thyroid s"[All Fields] OR "thyroidal"[All Fields] OR "thyroideal"[All Fields] OR "thyroidism"[All Fields] OR "thyroiditis"[MeSH Terms] OR "thyroiditis"[All Fields] OR "thyroiditides"[All Fields]) AND "cancer*"[All Fields]) OR (("thyroid gland"[MeSH Terms] OR ("thyroid"[All Fields] AND "gland"[All Fields]) OR "thyroid gland"[All Fields] OR "thyroid"[All Fields] OR "thyroid usp"[MeSH Terms] OR ("thyroid"[All Fields] AND "usp"[All Fields]) OR "thyroid usp"[All Fields] OR "thyroids"[All Fields] OR "thyroid s"[All Fields] OR "thyroidal"[All Fields] OR "thyroideal"[All Fields] OR "thyroidism"[All Fields] OR "thyroiditis"[MeSH Terms] OR "thyroiditis"[All Fields] OR "thyroiditides"[All Fields]) AND "gland*"[All Fields]) OR (("thyroid gland"[MeSH Terms] OR ("thyroid"[All Fields] AND "gland"[All Fields]) OR "thyroid gland"[All Fields] OR "thyroid"[All Fields] OR "thyroid usp"[MeSH Terms] OR ("thyroid"[All Fields] AND "usp"[All Fields]) OR "thyroid usp"[All Fields] OR "thyroids"[All Fields] OR "thyroid s"[All Fields] OR "thyroidal"[All Fields] OR "thyroideal"[All Fields] OR "thyroidism"[All Fields] OR "thyroiditis"[MeSH Terms] OR "thyroiditis"[All Fields] OR "thyroiditides"[All Fields]) AND "disease*"[All Fields]) OR (("thyroid gland"[MeSH Terms] OR ("thyroid"[All Fields] AND "gland"[All Fields]) OR "thyroid gland"[All Fields] OR "thyroid"[All Fields] OR "thyroid usp"[MeSH Terms] OR ("thyroid"[All Fields] AND "usp"[All Fields]) OR "thyroid usp"[All Fields] OR "thyroids"[All Fields] OR "thyroid s"[All Fields] OR "thyroidal"[All Fields] OR "thyroideal"[All Fields] OR "thyroidism"[All Fields] OR "thyroiditis"[MeSH Terms] OR "thyroiditis"[All Fields] OR "thyroiditides"[All Fields]) AND "neoplasm*"[All Fields])) AND ("diagnos*"[All Fields] OR "screen*"[All Fields] OR "assess*"[All Fields] OR "identif*"[All Fields]) |
| SCOPUS | ( TITLE-ABS-KEY ( "infrared thermography" OR "thermography" OR "thermal image" OR "thermogram" OR therm* OR "infrared therm*" OR "temperature mapping" ) ) AND ( TITLE-ABS-KEY ( "thyroid nodule*" OR "thyroid cancer*" OR "thyroid gland*" OR "thyroid disease*" OR "thyroid neoplasm*" ) ) AND ( TITLE-ABS-KEY ( "diagnos*" OR "screen*" OR "assess*" OR "identif*" ) ) |
| Web of Science | TS=(("infrared thermography" OR "thermography" OR "thermal image" OR "thermogram" OR “therm*” OR "infrared therm*" OR "temperature mapping")) AND TS=(("thyroid nodule*" OR "thyroid cancer*" OR "thyroid gland*" OR "thyroid disease*" OR "thyroid neoplasm*")) AND TS=(("diagnos*" OR "screen*" OR "assess*" OR "identif*")) |
| EMBASE | ('infrared thermography'/exp OR 'infrared thermography' OR 'thermography'/exp OR 'thermography' OR 'thermal image' OR 'thermogram'/exp OR 'thermogram' OR 'therm' OR 'infrared therm' OR 'temperature mapping') AND ('thyroid nodule'/exp OR 'thyroid nodule' OR 'thyroid cancer'/exp OR 'thyroid cancer' OR 'thyroid gland'/exp OR 'thyroid gland' OR 'thyroid disease'/exp OR 'thyroid disease' OR 'thyroid neoplasm'/exp OR 'thyroid neoplasm') AND ('diagnos' OR 'screen'/exp OR 'screen' OR 'assess' OR 'identif') |
| Google Scholar | thermogram, thyroid cancer, screening |
